# Thermally assisted self-healing behavior of anhydride modified polybenzoxazines based on transesterification

**DOI:** 10.1038/s41598-018-27942-9

**Published:** 2018-07-09

**Authors:** Feiya Fu, Meiqi Huang, Weilan Zhang, Yang Zhao, Xiangdong Liu

**Affiliations:** Key Laboratory of Advanced Textile Materials and Manufacturing Technology, Ministry of Education, College of Materials and Textile, Zhejiang Sci-Tech University, Xiasha Higher Education Zone, Hangzhou, 310018 P.R. China

## Abstract

A self-healing polybenzoxazine is synthesized solely based on dynamic ester bonds. For this purpose, an anhydride (succinic anhydride) was added into bisphenol F derived benzoxazine monomer before thermocuring. Owing to the transesterification of newly formed ester bonds, the thermoset network behaves as a thermoplastic at 140 °C in the presence of Zn (Ac)_2_, and shows self-healing properties even after multiple damage-healing cycles. Furthermore, kinetics study indicates that the transesterification is a first-order reaction and the activation energy is about 135.4 kJ/mol. This study proposes a facile and economical way to prepare self-healing polybenzoxazine. It has promising applications in coating, adhesive, and other smart materials that rely on structurally dynamic polymers.

## Introduction

Over the past few decades, there has been an explosive interest in developing self-healing materials because of their built-in ability to repair physical damage, effectively avoiding catastrophic failure and extending the working life^1,2^. According to the healing mechanisms, self-healing materials can be divided into types of extrinsic self-healing and intrinsic self-healing^3^. The former achieve their self-healing process through releasing healing agents from capsules. The latter is based on a dynamic, reversible linkage/reaction, such as reversible covalent bonds, metalligand interactions, and multiple hydrogen bonding^4,5^. Particularly, reversible interactions are of particular interest due to their capability to heal repeated damage at the same position^6^.

Recently, elegant dynamic covalent chemistries have been introduced in thermosetting polymers to yield self-healing or stress-relaxation properties^7,8^. In 2011, Montarnal and co-workers designed and realized covalently crosslinked epoxy networks that behaved like silica which could be malleable, reparable, and recyclable^9^. The underlying concept was to allow for reversible exchange reactions by transesterification. The chemistry was versatile, relied on readily available ingredients, and did not require any special equipment. Based on the theoretical study, a number of self-healing thermosetting polymers have been reported in recent years^10^. For example, Leibler et.al demonstrated the healing capability of epoxy-acid and epoxy-anhydride thermoset networks at 50 °C^7^. Lu et.al reported a shape memory and healable epoxy based on esterification between diglycidyl ether of bisphenol A and phthalic anhydride^11^.

Polybenzoxazine (PBZ) is a relatively new phenolic resin and has gained considerable attention in recent years^12^. Compared to conventional novolac and resole type phenolic resins, PBZ exhibits various outstanding characteristics, including high modulus and strength, chemical and thermal stabilities and near-zero shrinkage upon curing^13,14^. The synthesis of polybenzoxazines can simply be achieved by thermally activated ring-opening polymerization of its corresponding 1,3-benzoxazine monomers with or without a catalyst. The properties of polybenzoxazines resins can be regulated by various chemistries, which endow polybenzoxazines with a promising application in many fields^15–23^. However, synthesis of bulk polybenzoxazines with self-healing property has scarcely been investigated, though some polybenzoxazine precursor has been used as a self-healing additive^17,24,25^. Most recently, Mustafa Arslan *et al*. reported that self-healing PBZ thermoset could be achieved based on the supramolecular attraction^26^ and S-S bond cleavage-reformation reaction^27^. Yagci, Yusuf *et al*. prepared a self-healing poly(propylene oxide)-polybenzoxazine thermosets by photoinduced coumarine dimerization^28^. The performance of the resultant material was appealing but the always involved conventional main chain precursor synthesis methodology requiring multiple steps was somewhat complicated.

**Figure 1:**
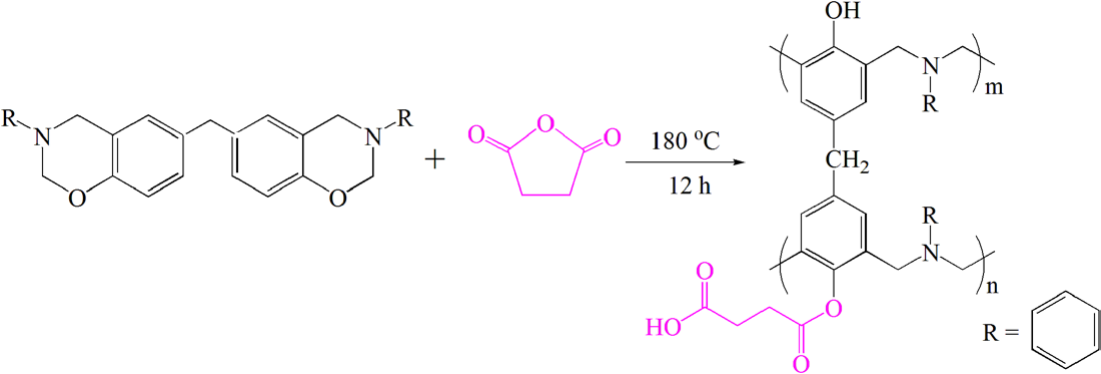
Synthesis of PBZ-SA thermoset from BZ monomer and SA.

Herein, we were interested in designing self-healing polybenzoxazines solely based on dynamic ester bond. Accordingly, a small amount of succinic anhydride was added into the benzoxazine monomers and ester bond form between the anhydride and phenolic hydroxyl groups after thermocuring (Fig. 1). The mechanical properties of the network were characterized by DMA and fatigue test. Additionally, the kinetics of transesterification was investigated using TGA method. The results illustrated that the synthesized polybenzoxazines could be reprocessed at higher temperature, and its capability to heal repeated damage at the same position could also be achieved.

## Results and discussion

**Figure 2:**
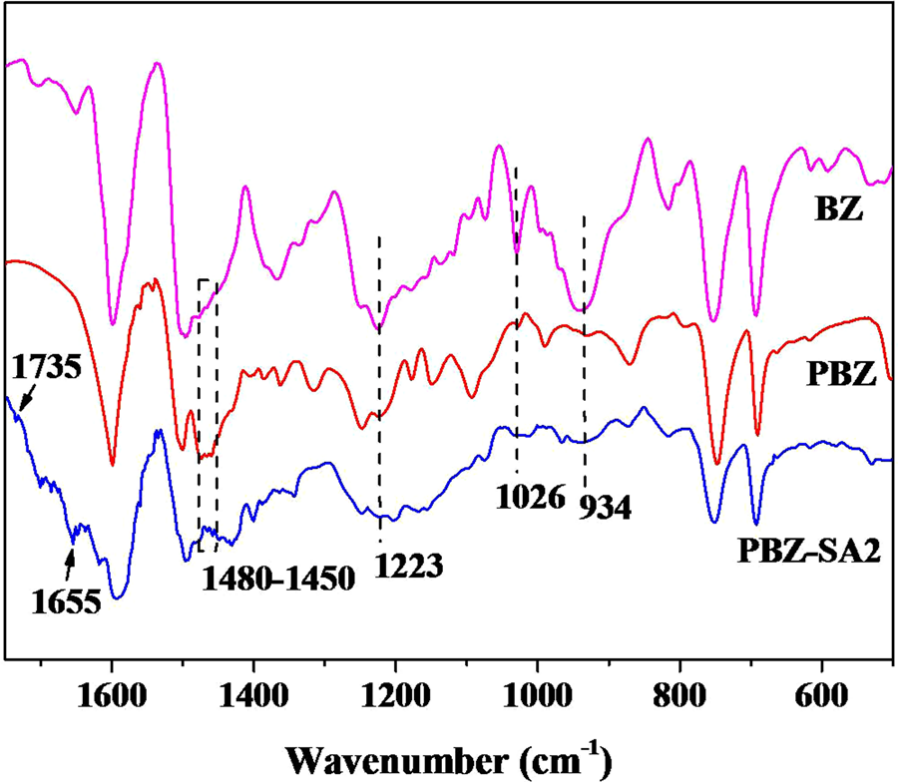
FT-IR spectra of the BZ monomer, pure PBZ and PBZ-SA2 thermoset.

The curing processes of the benzoxazine monomer/SA/Zn(Ac)_2_ mixtures were monitored by DSC at a heating rate of 10 °C min^−1^ from 25 °C to 300 °C. As shown in Fig. S1, the neat benzoxazine displays exothermic peaks in the temperature range from 180 °C to 240 °C, In contrast, the addition of SA and Zn(Ac)_2_ results in two exothermic peaks at lower temperatures, which suggested that complex reaction mechanisms were present and the curing process of the neat benzoxazine was promoted. Figure 2 shows the FT-IR spectra of PBZ-SA2 thermoset compared with pure PBZ and BZ monomer. The oxazine ring of BF showed three characteristic peaks at 934 cm^−1^, 1026 cm^−1^, and 1223 cm^−1^, assigned to the ring symmetric and anti-symmetric stretching of the C-O-C bond^29–31^. However, the three peaks in PBZ were almost disappeared completely, and characteristic peaks assigned to tetra-substituted benzene (1450–1480 cm^−1^) increased^32^. The results clearly demonstrated the ring-opening reaction of the BZ monomer^33^. With introduction of SA in PBZ network, new peaks attributed to the stretching vibration of the carbonyl (C=O) in the base of ester bond^34^ and Zn(Ac)_2_^35^ immerged at 1735 and 1655 cm^−1^, respectively. This illustrated that ester bond was formed between phenolic hydroxyl group of PBZ and SA after thermocuring.

**Figure 3:**
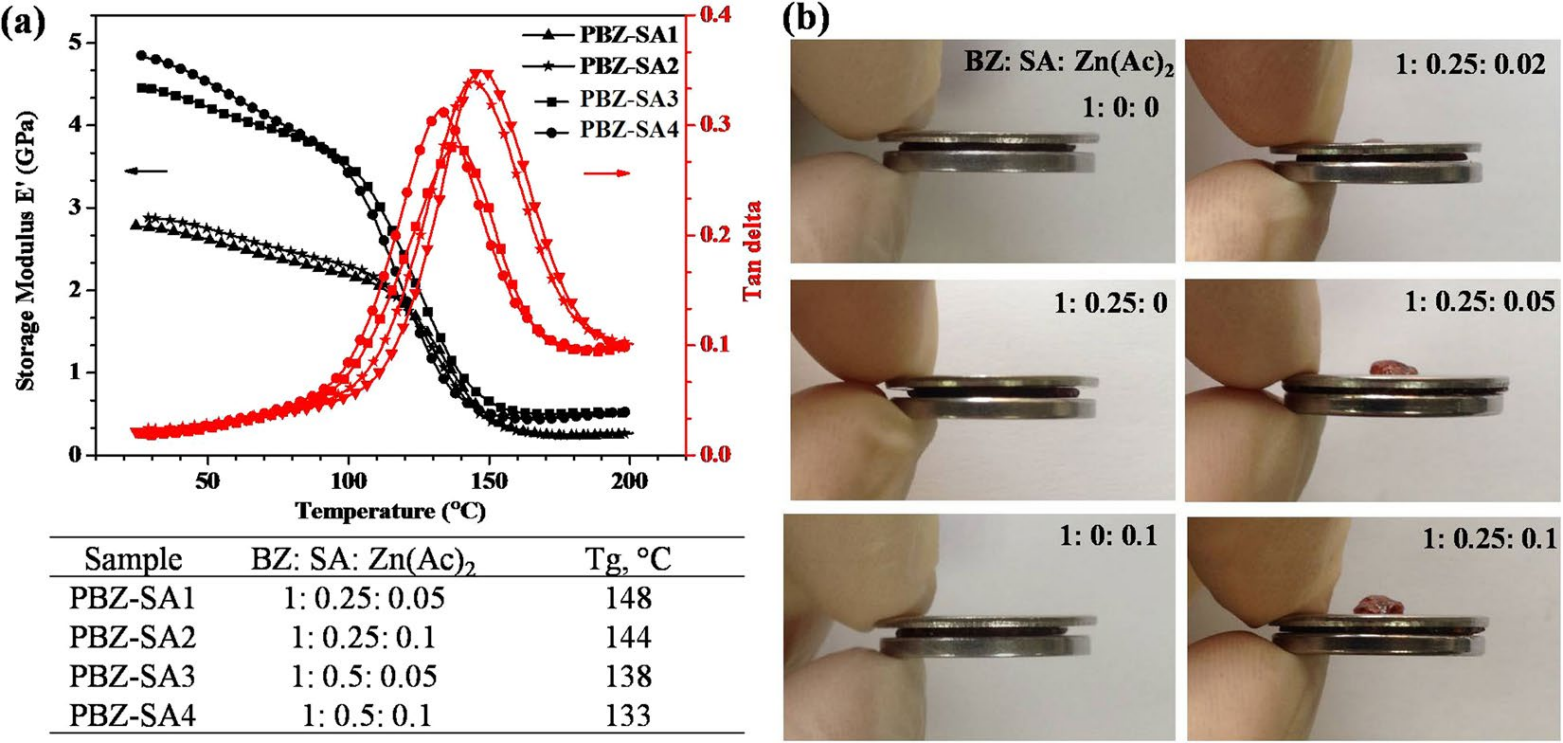
(**a**) spectra of PBZ-SA thermosets with different composition and (**b**) images of the thermosets after thermal deformation test.

Dynamic mechanical analyses were performed to measure the mobility of the chain segments in the network. As shown in Fig. 3a, the *E*′ values of PBZ-SA3 and PBZ-SA4 network with more SA was obvious higher than that of PBZ-SA1 and PBZ-SA2, especially at lower temperature (35–100 °C). On the contrary, the glass transition temperatures (Tg) of PBZ-SA3 (138 °C) and PBZ-SA4 (133 °C), which could be reflected by the tanδ peaks, were apparently lower than that of PBZ-SA1 (148 °C) and PBZ-SA2 (144 °C). One of possible reasons for this was that the crosslink density of the thermoset increased by adding SA^36^, however, more transesterification reaction would happen, which might improve the flexibility of the network simultaneously^37^. In addition, by only increasing the Zn(Ac)_2_ content, it was noted that the *E*′ of the PBZ-SA2 and PBZ-SA4 had no marked change when compared to PBZ-SA1 and PBZ-SA3, respectively, while the Tg values showed a slightly decrease. It could explained by that Zn(Ac)_2_ could only accelerate transesterification reaction while the total number of ester bonds remained unchanged^9^. Similarly, as shown Fig. 3b, obvious deformation was observed in all PBZ-SA samples (right column) after thermal deformation test, and the volume of overflow part increased with more Zn(Ac)_2_ was added. In sharp contrast, the PBZ thermoset (left column) would not flow for lack of SA or Zn(Ac)_2_.

**Figure 4:**
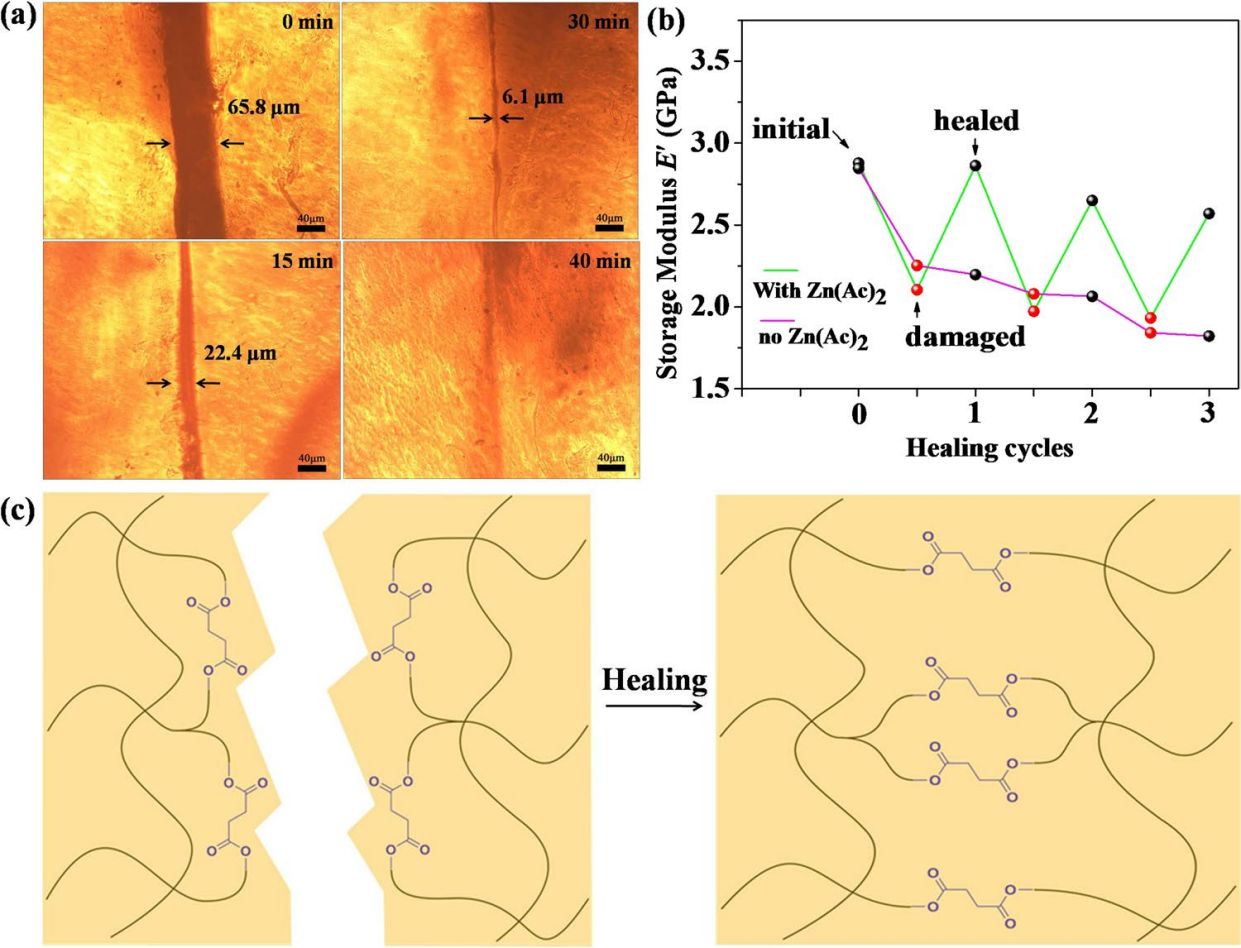
(**a**) Time-dependent change for the cut on the surface of PBZ-SA2 film, (**b**) storage modulus of PBZ-SA2 after the damage-healing cycle, (**c**) an illustration of the self-healing process for the PBZ-SA thermoset.

In order to demonstrate the self-repairing capability of the PBZ-SA, the optical micrographs of the cut and healed specimens was presented in Fig. 4a. Initially, a cut with 65.8 μm was found. With prolong of the healing time, the damaged area significantly decreased, and width of the damage came to 6.1 μm after 30 min. After heating at 140 °C for 40 min, the damage was almost totally healed. Further, as the ability of repeated recovery was a primary advantage for healable materials prepared via reversible bond exchange, the self-healing ability of the present thermoset network at a same site was investigated through multiple damage-healing cycles. As presented in Fig. 4b, the *E*′ values of PBZ-SA samples decreases from 2.88 GPa to 2.10 GPa after the first damage, but the loss could be regained mostly (2.86 GPa) after heating. The healing efficiencies of the PBZ-SA samples after one, two, and three healing cycles was 99%, 92% and 89%, respectively, when comparing *E*′ values of the healed sample to the original sample. In contrast, the PBZ-SA network without containing Zn(Ac)_2_ could not be repaired by healing, and its *E*′ values decreased linearly after damage. In the present work, as illustrated in Fig. 4c, the healing behavior arose from dynamic ester bonds present at the interface of a cut^10^. With the existence of Zn(Ac)_2_, the rate of ester bond breaking-reforming raised and the free phenolic hydroxyl and carboxyl group could form bridges with those on the adjacent surface to span the divide at the site of damage^38^.

**Figure 5:**
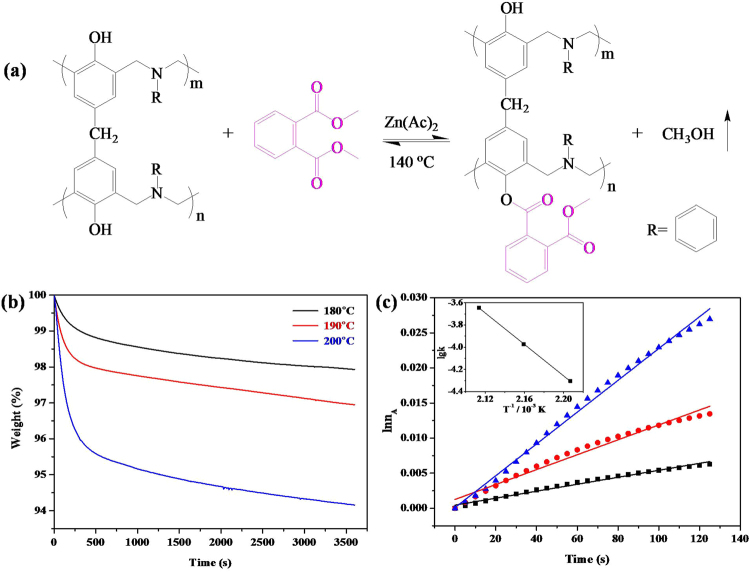
(**a**) The transesterification reaction between PBZ and DMP, (**b**) Isothermal TGA curves of PBZ-DMP mixture treated at different temperature. (**c**) The plots of ln(n_A_) versus the reaction time, inset is the plots of ln*k* against 1/T.

To study the kinetics of transesterification reaction in thermoset network, SA was replaced by dimethyl phthalate (DMP), and the reaction rate could be determined by calculating the amount of released methyl alcohol during the isothermal TGA test (Fig. 5a). As shown in Fig. 5b, the weight of PDP-BZ kept dropping with ongoing of heating at 180 °C. This implied that the transesterification reaction did occur in the system.^39^ Moreover, the weight loss of the system was more remarkable at 190 and 200 °C, indicating an increase of transesterification reaction rate. Additionally, it was noted that natural logarithm of n_*a*_ (mole number of methyl alcohol) yield a linear relationship with the reaction time for all the conditions (Fig. 5c). This suggested that transesterification reaction between phenolic hydroxyl of PBZ and DMP could be treated as a first-order reaction^40^. According to the integrated Arrhenius equation^41^:

1 $$\mathrm{ln}\,k=-\,\frac{{E}_{a}}{R}\ast \frac{1}{T}+\,\mathrm{ln}\,A$$

A plot of ln*k* versus 1/T should result in a linear relationship with the slope equal to −*E*_a_/R. As illustrated by the Arrhenius plots in Fig. 5c (inset), the two variables shows a good linear relationship (correlation coefficient, 0.986) and the activation energy was calculated to be 135.4 kJ/mol.

## Conclusion

In conclusion, based on the strategy of dynamic ester bonds, a new self-healing polybenzoxazine was synthesized through reactions between succinic anhydide and phenolic hydroxyl group of polybenzoxazine. The self-healing properties of the network were observed by optical microscopy, DMA and fatigue test. The results illustrated it was possible to achieve bulk-state self-healing in the polybenzoxazines thermoset via ester bond exchange. Particularly, in the presence of accelerator (Zn(Ac)_2_), the dynamic covalent nature of the bonds allowed healing to occur over multiple cycles. Furthermore, the TGA test indicates that the transesterification reaction could be treated as a first-order reaction and the activation energy was estimated to be 135.4 kJ/mol. Through this study, it was clear that introduction of dynamic ester bond did promise a successful approach to achieve self-healing polybenzoxazines, which could further expanding its use in high performance materials.

## Methods

### Materials

Bisphenol F based benzoxazine (BZ, 75 wt% in butanone) was obtained from Huntsman investment Co., Ltd (Utah, USA). succinic anhydride (SA), zinc acetate (Zn(Ac)_2_) and dimethyl phthalate (DIP) were purchased from Aladdin Reagent Co. (Shanghai, China). The reagents and solvents were used as received.

### Synthesis of self-healing polybenzoxazines thermoset

BZ, SA, and Zn(Ac)_2_ were mixed at 40 °C and degassed using a conditioning mixer (AR-100, Thinky, Japan). Then, the resulting mixture was dried at 60 °C under vacuum to remove butanone, and thermocured at 180 °C for 12 h. By changing the mole ratio of the BF/SA/Zn(Ac)_2_ mixture from 1:0.25:0.05, 1:0.25:0.1, 1:0.5:0.05 to 1:0.5:0.1, the obtained thermoset was coded as PBZ-SA1, PBZ-SA2, PBZ-SA3 and PBZ-SA4, respectively. FTIR (Nicolet Company, Madison, USA), and DMA (Q800 DMA, USA) were used to characterize the thermoset network. To reduce the errors generated in the forming process, the BZ/SA/Zn(AC)_2_ mixtures were cured to composite samples with standard filter paper (pore size of 18 mm) as a reinforcement filler for all the DMA tests.

### Synthesis of PBZ-DMP thermoset

To study the kinetics of transesterification reaction, SA was replaced by DMP and a PBZ-DMP thermoset was prepared through a process similar to the self-healing polybenzoxazines. The molar ratio of BF/DMP/Zn(Ac)_2_ mixture was set as 1:0.25:0.1 and the transesterification was monitored using a isothermal TGA (TGA/SDTA851, Switzerland) method.

### Measurements

Thermal deformation test was performed as follows: the cured thermoset piece was placed between two metal pads, and one of the pads has a round opening (diameter, 20 mm). Then, the pads were clamped by two binder clips with a force of ~10 N, and heated at 140 °C (30 min) for observation.

The multiple self-healing ability of the thermoset at a same site was performed on a fatigue machine (SJY-500, Shandu, China).The three-point bending method was applied, with a loading force of 25 N at a frequency of 3 Hz (Fig. S2). During each healing cycle, the sample was firstly subjected to 2000 times of impact breakage on the fatigue machine, and then healed by heat treatment at 140 °C for 1 h. The storage modulus (*E*′) of the recycled thermosets was determined using the DMA machine.

## Electronic supplementary material

Supplementary Information
