# Supplementary material for: Thermally assisted self-healing behavior of anhydride modified polybenzoxazines based on transesterification
Source: Sci Rep. 2018 Jul 9;8:10325. doi: 10.1038/s41598-018-27942-9 (PMC6037739; doi:10.1038/s41598-018-27942-9)
Supplement: Supplementary file 1 — Supplementary Information [file 41598_2018_27942_MOESM1_ESM.pdf]

Thermally assisted self-healing behavior of anhydride modified polybenzoxazines based on transesterification

Feiya Fu[1], Meiqi Huang[1], Weilan Zhang[1], Yang Zhao[1] & Xiangdong Liu[1]

[1] Key Laboratory of Advanced Textile Materials and Manufacturing Technology, Ministry of Education, College of Materials and Textile, Zhejiang Sci-Tech University, Xiasha Higher Education Zone, Hangzhou 310018, P.R. China. Correspondence and requests for materials should be addressed to X.D.L. (email: liuxd@zstu.edu.cn)

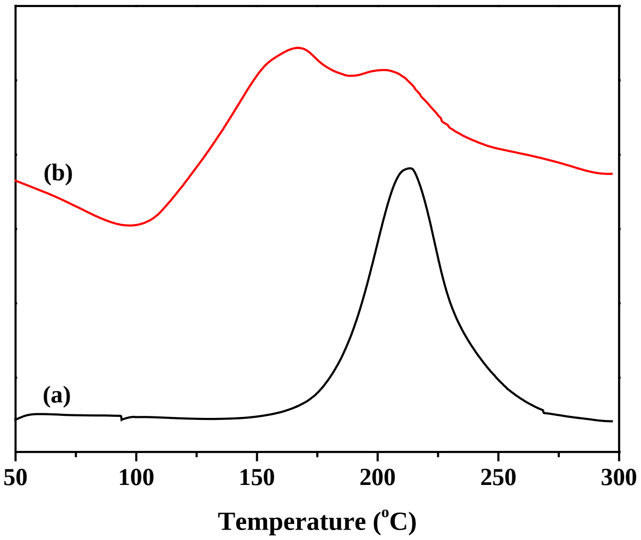

Supplementary Figure S1. DSC thermograms of the BZ monomer and the mixture (BZ monomer, succinic anhydride and $Zn(Ac)_2$).

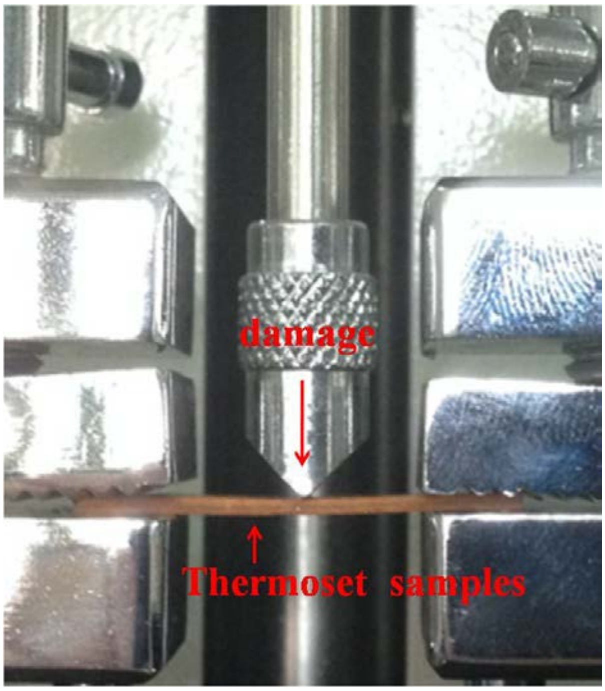

Supplementary Figure S2. Picture of the fatigue machine used for evaluating self-healing ability of the thermoset samples through three-point bending method.
